# Short-Term Interactions of *Noctiluca scintillans* with the Toxic Dinoflagellates *Dinophysis acuminata* and *Alexandrium minutum*: Growth, Toxins and Allelopathic Effects

**DOI:** 10.3390/toxins15060373

**Published:** 2023-06-01

**Authors:** Soledad Garrido, Pilar Riobó, Pilar Rial, Francisco Rodríguez

**Affiliations:** 1 Spanish Institute of Oceanography, Spanish National Research Council, 36390 Vigo, Spain; garridosoledad21@gmail.com (S.G.); pilar.rial@ieo.csic.es (P.R.); 2 Instituto de Investigaciones Marinas, Spanish National Research Council, 36208 Vigo, Spain; pilarriobo@iim.csic.es

**Keywords:** *Noctiluca scintillans*, *Alexandrium minutum*, *Dinophysis acuminata*, toxins, diarrhetic shellfish poisoning, paralytic shellfish poisoning, HPLC, HAB, allelopathy

## Abstract

The Galician Rías (NW Iberian Peninsula) are an important shellfish aquaculture area periodically affected by toxic episodes often caused by dinoflagellates such as *Dinophysis acuminata* and *Alexandrium minutum*, among others. In turn, water discolorations are mostly associated with non-toxic organisms such as the heterotrophic dinoflagellate *Noctiluca scintillans*, a voracious non-selective predator. The objective of this work was to study the biological interactions among these dinoflagellates and their outcome in terms of survival, growth and toxins content. To that aim, short experiments (4 days) were carried out on mixed cultures with *N. scintillans* (20 cells mL^−1^) and (i) one strain of *D. acuminata* (50, 100 and 500 cells mL^−1^) and (ii) two strains of *A. minutum* (100, 500 and 1000 cells mL^−1^). Cultures of *N. scintillans* with two *A. minutum* collapsed by the end of the assays. Both *D. acuminata* and *A. minutum* exposed to *N. scintillans* arrested its growth, though feeding vacuoles in the latter rarely contained any prey. Toxin analyses at the end of the experiment showed an increase in intracellular OA levels in *D. acuminata* and a significant reduction in PSTs in both *A. minutum* strains. Neither OA nor PSTs were detected in *N. scintillans*. Overall, the present study indicated that the interactions among them were ruled by negative allelopathic effects.

## 1. Introduction

*Noctiluca scintillans* (Macartney) Kofoid & Swezy is a common heterotrophic dinoflagellate species with worldwide distribution, e.g., [1,2,3]. In favorable conditions, its trophont populations originate blooms that end up accumulating in surface layers, causing seawater discolorations (so-called red tides) [4]. These surface patches show typical concentrations between 100 and 3000 cells mL^−1^ and look reddish if they belong to the red form (in contrast with the green *N. scintillans* variety mostly restricted to tropical Asian waters) [3]. The development of *N. scintillans* proliferations usually takes place in inshore coastal areas with large nutrient inputs, both from natural or anthropogenic sources, such as estuaries and upwelling areas [3,4,5]. These environments allow the development of phytoplankton required for their growth (e.g., diatoms and dinoflagellates), which stands as the obligate source of energy in red *N. scintillans*.

Although *N. scintillans* does not produce any toxins, it is categorized as a harmful species due to the high levels of ammonium accumulated inside the cells and oxygen depletion during the decay of high-abundance events [6]. In fact, blooms of *N. scintillans* have been reported to cause mortalities in caged fish and its ammonia-containing vacuoles have been implicated [7].

Previous studies have shown that *N. scintillans* is a voracious non-selective predator, with its diet ranging from phytoplankton (mainly diatoms, e.g., [8]) to ciliates and copepod eggs [9]. Predation by heterotrophic protists such as *N. scintillans* is one of the causes of mortality in marine phytoplankton [10]. Given that *N. scintillans* does not discriminate between toxic and non-toxic prey, its potential role as a vector of marine biotoxins in the food web has been suggested [11], from mainly exerting top-down control during most of its pelagic life to fueling bottom-up processes due to the liberation of intracellular nutrients during senescence [12]. It may also have some influence on the population dynamics of harmful algae [13]. This can be especially intense during the massive summer blooms of *N. scintillans*.

The coastal upwelling system of the NW Iberian Peninsula is characterized by natural flooded tectonic valleys called “Rías” [14], commonly affected by HABs and red tides caused, among other groups, by dinoflagellates [15,16]. In fact, environmental conditions, particularly in the summer months, are suitable for the periodical development of dinoflagellates, often including toxic species from the genera *Alexandrium* and *Dinophysis* [17,18]. These organisms and their HABs can be present during the same period where red tides of *N. scintillans* take place, such as in September 2021 when extensive orangish surface patches due to this species were observed in two Rías (Pontevedra and Vigo) [19]. The bioaccumulation of toxins in the trophic food web gives rise to significant socioeconomic damage due to shellfish harvesting closures and the intensive aquaculture industry in the region [20,21]. Thus, as explained before, the proliferation of *N. scintillans* in the Rías, and the potential effects of their populations on HAB species, raises intriguing ecological questions regarding their impact on the community and the potential transfer of toxins. In the case of toxic phytoplankton, a defensive mechanism against grazers and competitors is the most plausible hypothesis for the evolution of their toxins and the negative allelopathic effects played by these and other secondary metabolites [22]. Nevertheless, few studies have examined this issue in mixed cultures of *N*. *scintillans* [8,9,13].

The contribution of biotic interactions, either positive (mutualism, commensalism) or negative (antagonism, predation, parasitism) [23,24], to the onset, duration and intensity of harmful algal blooms (HABs) has often been overlooked because of the difficulty of detecting them. HABs involve a wide diversity of organisms with distinct population dynamics and mechanisms of impact [4]. Toxic effects and other deleterious consequences from HABs may involve several taxonomic groups [25,26], dinoflagellates being the most relevant—at least in terms of toxin production [27].

Allelochemicals are often related with benefits (i.e., through facilitating access to resources, inducing damage to protist competitors and defensive mechanisms) [26]. Thus, the consequences of the biotic interactions mediated by toxins and these compounds could contribute to explaining the ecological success of some dinoflagellates and HAB species in the field.

In the present work, the biotic interactions between *N. scintillans* and two representative toxic dinoflagellates originating from the same area, *Dinophysis acuminata* and *Alexandrium minutum*, were examined. *D. acuminata* is responsible for a high proportion of HABs and shellfish harvesting closures in the Rías (and Western Europe) due to the accumulation of lipophilic shellfish toxins (okadaic acid (OA), dinophysistoxins (DTXs) and their congeners [25,28]. *A. minutum* represents historically one of the main paralytic shellfish toxin (PST) producers in the Rías, with variable toxin profiles and potential impacts on human health and aquaculture activities (despite a few records of non-toxic populations [29]). Our aim was to study their short-time evolution and detect negative allelopathic effects from these potential prey in mixed cultures, as well as following the evolution in toxicity and toxin contents during the experiments.

Results from the present work could help in understanding the allelopathic effects of *N. scintillans* over HAB species and the consequences of the biotic interactions between these organisms which coexist during the growth season. To that purpose, cells isolated from a red tide of *N. scintillans* in summer 2021 were exposed to different levels (up to bloom densities) of these two toxin-producing dinoflagellates.

## 2. Results

The presence of prey inside the feeding vacuoles of *N. scintillans* was rarely observed during the assays with *Dinophysis* and *Alexandrium* strains. The effects of two factors (exposure time and cell abundance) on the evolution of mixed cultures of *N. scintillans* with either *D. acuminata* (VGO1465) or *A. minutum* (VGO1435 and VGO1439) were examined. Ingestion and clearance rates were not determined in this work and only the evolution of the studied organisms was measured by cell counts in the controls and mixed cultures.

### 2.1. Dinophysis acuminata VGO1465 Assay 

**Figure 1:**
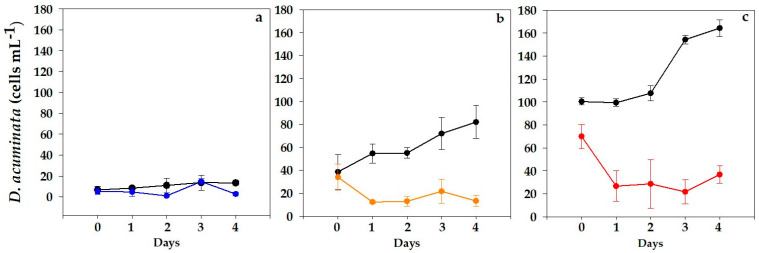
Evolution of *D. acuminata* (VGO1465) cell counts in control (black line) and mixed cultures with *N. scintillans* (20 cells mL^−1^). Initial concentrations: (**a**) 10 cells mL^−1^ (blue line), (**b**) 50 cells mL^−1^ (orange line) and (**c**) 100 cells mL^−1^ (red line). Data represent mean (*n* = 3) and error bars correspond to the SD.

Three cell densities of *D. acuminata* (10, 50 and 100 cells mL^−1^) were exposed to cultures of *N. scintillans* (20 cells mL^−1^), and daily variations in *D. acuminata* cell counts are shown in Figure 1. A slight increase in *D. acuminata* was observed in the lowest concentration (10 cells mL^−1^; Figure 1a), but no significant differences were found between the control and mixed cultures.

Steady growth was observed in *D. acuminata* controls in the intermediate concentration (50 cells mL^−1^; Figure 1b), while those exposed to *N. scintillans* significantly decreased during the first day (*p* < 0.001) then remained invariable over the next days.

The results obtained for the highest concentration (100 cell mL^−1^; Figure 1c) followed similar trends. At this level, the initial cell densities of *D. acuminata* were somewhat lower in mixed cultures in comparison with controls. Steady positive growth was apparent in *D. acuminata* control replicates throughout the bioassay, with maximum specific growth rates of 1.93 d^−1^ between days 2 and 4. On the contrary, *D. acuminata* exposed to *N. scintillans* exhibited a significant decrease (*p* < 0.001) throughout the incubation period.

**Figure 2:**
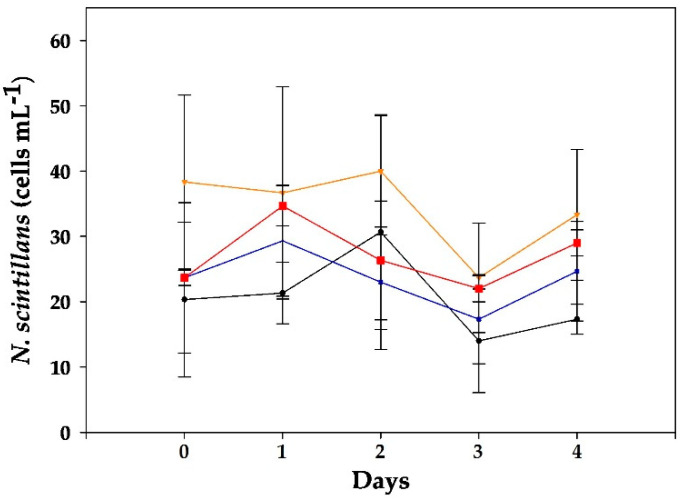
Evolution of *N. scintillans* cell counts in control (black line) and experimental treatments exposed to three initial concentrations of *D. acuminata*: (blue) 10 cells mL^−1^, (orange) 50 cells mL^−1^ and (red) 100 cells mL^−1^. Data represent mean (*n* = 3), and error bars correspond to the SD.

Meanwhile, in the case of *N. scintillans*, rather constant cell densities were observed in control and mixed cultures, only with slightly lower abundances by the end of the experiment (Figure 2).

#### OA Analyses

The studied strain of *D. acuminata* produced only OA. The lipophilic toxin results on one sample of the strain before the experiment yielded 27.04 pg OA cell^−1^ and 3.51 ng extracellular OA mL^−1^. Toxin analyses performed on *D. acuminata* controls and mixed cultures at the end of the experiment (day 4) showed higher intracellular OA and lower extracellular OA levels relative to the original culture (Table 1). No significant statistical differences between control and mixed cultures were obtained. In the case of *N. scintillans*, no detectable amounts of OA were determined.

### 2.2. Alexandrium minutum Assay

#### 2.2.1. VGO1435 Strain

**Figure 3:**
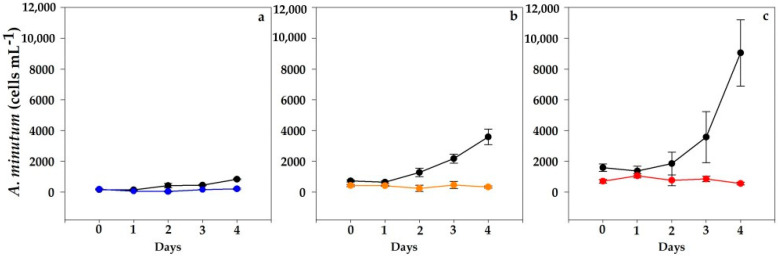
Evolution of *A. minutum* (VGO1435) cell counts in control (black line) and mixed cultures (blue, orange and red) throughout the assay. Initial concentrations: (**a**) 100 cells mL^−1^, (**b**) 500 cells mL^−1^ and (**c**) 1000 cells mL^−1^. Data represent mean (*n* = 3) and error bars correspond to the SD.

The control replicates of *A. minutum* at the three cell concentrations assayed (100, 500 and 1000 cells mL^−1^) showed similar high specific growth rates (4.6 ± 0.66 d^−1^; *n* = 9). Positive growth in controls sharply contrasted with invariable cell abundances in mixed cultures during the assay (Figure 3). The initial cell densities of *A. minutum* in mixed cultures at 1000 cells mL^−1^ were somewhat lower than in controls (Figure 3c).

The three mixed cultures of *A. minutum* (VGO1435) arrested their growth, with steady or lower concentrations on day 4. The differences between controls and mixed cultures were significant (*p* = 0.028–0.0001) from days 2 to 4. Few *A. minutum* cells could be seen inside *N. scintillans*’ feeding vacuoles, though many thecae were observed in the medium.

**Figure 4:**
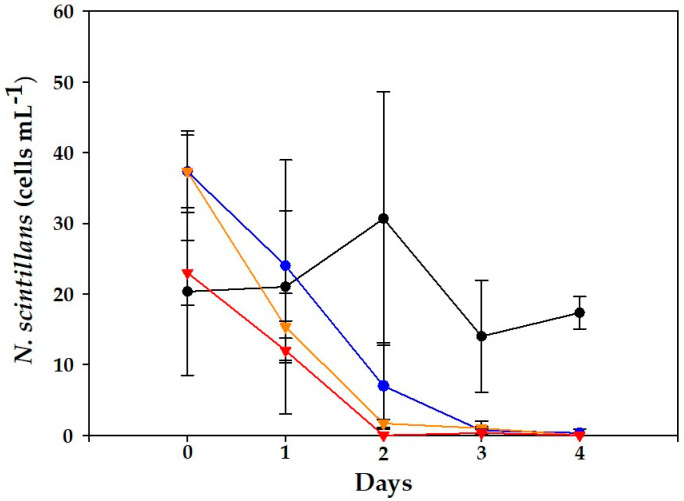
Evolution of *N. scintillans* cell counts for the control (black line) and cultures of *N. scintillans* exposed to *A. minutum* (VGO1435) during the experiments. Initial concentrations: (blue) 100 cells mL^−1^, (orange) 500 cells mL^−1^ and (red) 1000 cells mL^−1^. Data represent mean (*n* = 3) and error bars correspond to the SD.

Regarding the abundance of *N. scintillans*, significant differences were obtained between controls and mixed cultures (*p* < 0.01). In controls, until day 2, a slight growth could be noticed (1.9 d^−1^), and thereafter cultures declined to initial cell levels at day 4 (Figure 4). In contrast, all mixed cultures showed negative trends, which were statistically significant relative to the controls (days 0–2; *p* < 0.03), and collapsed between days 2 and 4.

##### PST Analyses

Paralytic toxins before the experiment were determined in one sample of the strain VGO1435 of *A. minutum* yielding 0.787 pg STX equivalents cell^−1^ (molar percentages: 91% GTX4; 6% GTX1; 2% GTX3; 1% GTX2). Toxin analyses were again performed on *A. minutum* controls and mixed cultures at the end of the experiment (day 4). PSTs were quantified in controls and higher toxin concentrations were measured in comparison with the original culture (Table 2). The toxin profile was characterized by the following compounds (ordered in decreasing relative abundance): GTX4, GTX3, GTX1 and GTX2. In turn, mixed cultures showed toxin levels below the LOD (of the analytical method applied) for every compound. No PSTs or dissolved toxins were found in *N. scintillans* or in the filtrate.

#### 2.2.2. VGO 1439 Strain

**Figure 5:**
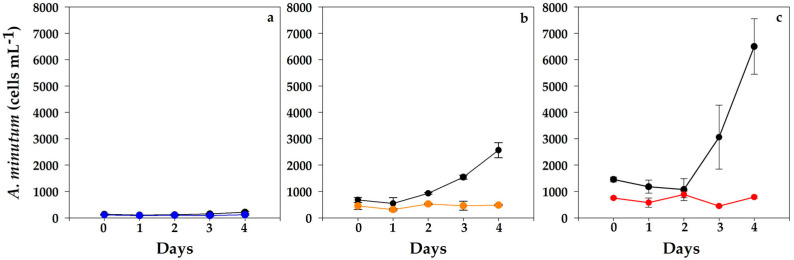
Evolution of *A. minutum* (VGO1439) cell counts of controls (black line) and experimental treatments assayed: (**a**) 100 cells mL^−1^ (blue line), (**b**) 500 cells mL^−1^ (orange line) and (**c**) 1000 cells mL^−1^ (red line). Data represent mean (*n* = 3) and error bars correspond to the SD.

In the case of *N. scintillans* exposed to the strain VGO1439 of *A. minutum*, the effect of cell abundance was the most statistically significant parameter for the final results (Figure 5), (*p* < 0.001, F = 324.787) (exposure time F = 21.57). In addition, *N. scintillans* cultures were exposed to a cell-free filtrate of VGO1435 *A. minutum* to discern any effects which could be associated with released compounds in the medium. In this case, the incubation time had the greatest statistical influence on the final results (F = 26.6) (experimental treatment F = 10.15).

From day 2 onwards, an exponential growth of *A. minutum* was observed in all controls. The growth rates attained were 4.4 d^−1^ and 5.3 d^−1^ in the experiments initiated with 500 and 1000 cells mL^−1^, respectively. Meanwhile, only a slight growth was recorded in the mixed cultures at the lowest abundance (Figure 5a; 100 cells mL^−1^). In contrast, the 500 and 1000 cells mL^−1^ mixed cultures did not grow at all (Figure 5b,c). The initial cell densities of *A. minutum* in mixed cultures at 1000 cells mL^−1^ were somewhat lower than in controls (Figure 5c). Significant differences (*p* < 0.001) were found with regard to the controls, especially on days 3–4. Overall, the final amounts of *A. minutum* remained similar to the initial values (Figure 5a–c).

**Figure 6:**
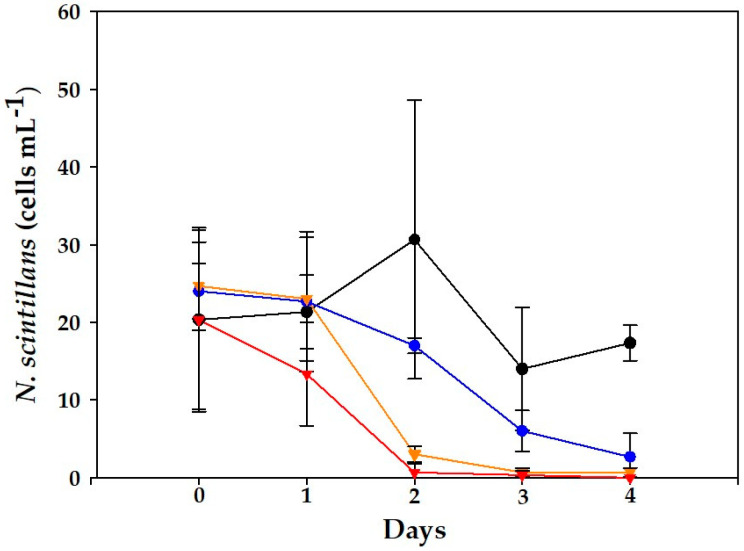
Evolution of *N. scintillans* cell counts for the control (black line) and mixed cultures exposed to *A. minutum* (VGO1439) during the experiment. Initial concentrations: (blue) 100 cells mL^−1^, (orange) 500 cells mL^−1^ and (red) 1000 cells mL^−1^. Data represent mean (*n* = 3) and error bars correspond to the SD.

Significant differences (*p* < 0.001) were observed between controls and mixed cultures in *N. scintillans* on days 3–4 (Figure 6). In the controls, until day 2, a slight increase was observed (1.9 d^−1^), but cell abundances decreased afterwards down to 14 ± 7.9 and 17 ± 2.3 cells mL^−1^. In addition, many empty thecae of *A. minutum* were observed in the medium, the same as with the VGO1435 strain.

In all experiments, *N. scintillans* exhibited a marked decrease in cell abundance. In fact, cell lysis in *N. scintillans* since day 2 was evident and proportional to the *A. minutum* cell concentrations.

**Figure 7:**
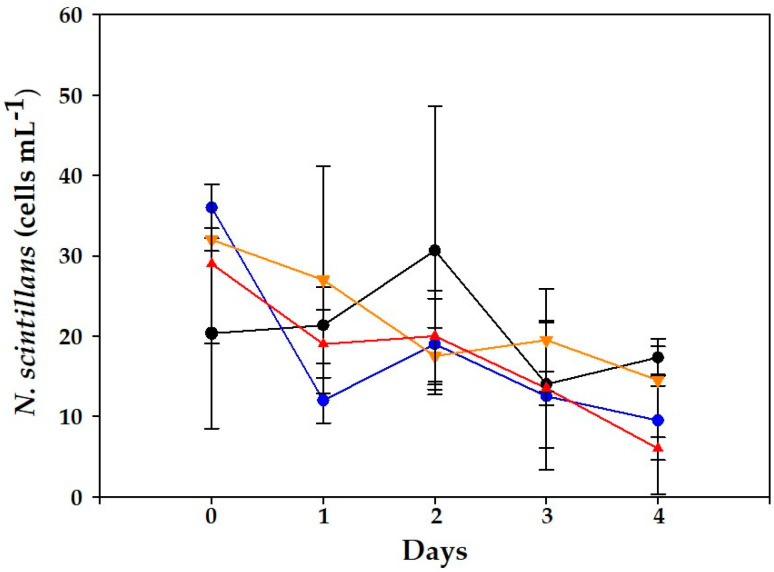
Evolution of *N. scintillans* cell counts for the control (black line) and mixed cultures with *A. minutum* (VGO1439) culture filtrate. Initial concentrations: (blue) culture filtrate from 100 cells mL^−1^, (orange) 500 cells mL^−1^ and (red) 1000 cells mL^−1^. Data represent mean (*n* = 3) and error bars correspond to the SD.

In the second (cell-free filtrate) experiment where *N. scintillans* was only exposed to culture filtrate from VGO1439, no significant differences were obtained between controls and mixed cultures (Figure 7). Nevertheless, in 500 and 1000 cells mL^−1^ there were significant differences (*p* < 0.001) in the number of surviving *N. scintillans* cells between this experiment and that with mixed cultures. This would explain why the survival of *N. scintillans* exposed to these filtrates after 4 days was slightly higher at the medium filtrate level. In this experiment, the incubation time had the greatest statistical influence (F = 26.6) (experimental treatment F = 10.15).

##### PST Analyses

A toxin analysis carried out before the experiment, in one sample of *A. minutum* (VGO1439), yielded 0.25 pg STX equivalents cell^−1^ (molar percentage: 94% GTX4; 6% GTX3). The toxin analysis on *A. minutum* controls and mixed cultures on day 4 of the experiment (Table 3) showed the highest PSTs levels in controls compared to the mixed cultures. There were significant differences in GTX4 between controls and mixed cultures in the 500 and 1000 cells mL^−1^ assays, especially in the former (*p* = 0.002). GTX4 was the dominant compound followed by GTX3. In mixed cultures, only GTX4 and GTX1 could be quantified. Most other compounds were <LOD due both to the lower toxin contents and final abundance of harvested cells on day 4. No PSTs were found in the culture filtrate or in *N. scintillans* cells.

## 3. Discussion

In the laboratory, *N. scintillans* cultures (its red form) have been maintained long term using the chlorophyte *Dunaliella salina* as prey [30]. Additionally, Fukuda et al. [4] employed another chlorophyte (*D. tertiolecta*) to detail the life cycle of *N. scintillans*. The evolution of *N. scintillans* cultures with different microalgal prey has been examined in a number of studies [12,30,31,32], including toxin-producing dinoflagellates [32,33,34]. However, the fate of marine biotoxins in plankton communities during the buildup of proliferations by *N. scintillans*, and the interactions between toxic prey and this organism, remains fairly under-investigated.

This study reports the first results for mixed cultures of *N. scintillans* and *D. acuminata* (Figure 2), one of the main producers of DSTs in NE Atlantic waters. An increase in the intracellular toxins of *D. acuminata* exposed to *N. scintillans* (Table 1) was observed, though the lack of statistical support prevented the establishment of a causative link. Nonetheless, this suggests that toxin production in this species could be upregulated in the presence of grazer cues. This possibility would deserve further exploration given its implications in toxic outbreaks of *D. acuminata*. This defensive mechanism has been proven in other toxic dinoflagellates and diatoms, e.g., *Alexandrium* and *Pseudo-nitzschia*; [9,35,36]. Moreover, micromolar concentrations of DSTs have been observed to inhibit growth in microalgae [37]. In turn, in the current study, no effects were observed on *N. scintillans* (neither in cell counts or its general aspect, viability, etc.) when exposed to *D. acuminata*, but it did arrest its growth, and the fact that its vacuoles rarely contained any prey suggests that negative allelopathic effects could play a major role in these results. The observation of lower initial cell densities in mixed cultures relative to controls at the highest level could not be explained by errors during cell manipulation as this was also registered in the *A. minutum* assays. Rather, it seemed to obey some other factor in these experiments (i.e., a higher initial grazing activity).

To confirm the observed trends in the *D. acuminata* assays regarding toxins, grazing, etc., further surveys including distinct strains and other commonly observed *Dinophysis* species (e.g., *D. acuta*, *D. caudata* and *D. fortii*) should be performed. The lowest initial cell densities of *D. acuminata* (10 cells mL^−1^) followed similar trends in control and mixed cultures, in contrast to those observed in the intermediate and high levels. This observation could be explained by the short-term duration of the experiment and the fact that a longer lag phase and slower initial growth are expected in batch cultures [38]. In turn, the faster recovery of growth with larger inoculum sizes allowed us to unmask the negative allelopathic effects with higher initial cell densities.

Indeed, this was also in the case of *A. minutum*. However, unlike *D. acuminata*, both studied strains induced lytic effects on *N. scintillans*. PSTs could play some role in the deleterious effects observed in *N. scintillans*. In contrast to the result observed in our *D. acuminata* experiments, toxin contents in *A. minutum* (Table 3) exposed to *N. scintillans* were lower than in control cultures. This result was also found by Azanza et al. [39] with another PST producer (*Pyrodinium bahamense*) actively grazed by *N. scintillans*.

Previous studies on the PST producer *A. catenella* [40,41] supported the idea that an increase in ammonia concentrations (as expected in mixed cultures with *N. scintillans*) also brings higher intracellular toxin contents. In this sense, *A. minutum* strains from the Rías Baixas study area showed reduced toxin contents under N-stress conditions [42]. However, we cannot ascertain the relationship between PSTs and the availability of N sources in the present work given that those were not determined. Nonetheless, regarding the outcome of growth experiments, it is unlikely that *N. scintillans* cells could yield ammonia levels high enough to inhibit dinoflagellate growth [43].

Frangópulos et al. [34] observed that the survival of *N. scintillans* exposed to various levels of *A. minutum* (50–400 cells mL^−1^) was >75%, without differences in toxin contents during the experiment (5 days). Thus, other compounds than PSTs, such as lytic exudates by *A. minutum*, could explain the observed mortality of *N. scintillans* in a density-driven manner found in our work (Figure 4 and Figure 6). These were particularly harmful above 500 cells mL^−1^, which corresponds with bloom densities in the field [21]. In the case of *Alexandrium*, it is known that most species produce bioactive extracellular compounds (BECs) which cause lytic effects on diverse organisms ranging from protists to mammalian cells [26]. BECs from *A. catenella* and *A. minutum* have been studied more in depth and include unknown large amphipathic compounds [26].

In the present study, harmful effects seemed to occur in both directions between *N. scintillans* and both *A. minutum* strains, but only from *N. scintillans* to *D. acuminata*. In this sense, the production of BECs or harmful effects has been little explored in laboratory cultures of *Dinophysis*. This is likely due to their limited availability and ease of cultivation in comparison with *A. minutum*. Therefore, to the best of our knowledge, negative allelopathic effects have only been shown by Mafra et al. [44] in *Dinophysis* cf. *ovum* affecting its ciliate prey *M. rubrum* (reduced cell abundance and “mucus traps” arresting their swimming). Interestingly, these authors revealed that other compounds than DSTs would be responsible for the observed harmful effects, possibly free PUFAs.

Previous studies exposing *N. scintillans* to distinct PST producers described similar outcomes of grazer–prey interactions. For instance, Stauffer et al. [33] observed that *N. scintillans* did not grow or even reduce its abundance when exposed to different levels of *A. catenella.* Hallegraeff et al. [32] exposed *N. scintillans* to cell-free *A. minutum* culture medium and stated this lytic effect, though toxin levels in the medium were not assessed. In our study, PSTs could not be detected in the medium and no significant differences were observed between controls and cell experiments (*p* > 0.9) when *N. scintillans* were exposed to cell-free culture filtrates (Figure 7). Other PST producers, such as *Gymnodinium catenatum*, have been shown to maintain stable growth rates of *N. scintillans* (0.3–0.60 d^−1^) for long periods (up to 8 months), but beyond a certain threshold (> 500 cells mL^−1^) they overwhelmed *N. scintillans* cultures [7].

Lastly, some experimental artifacts during the manipulation of *N. scintillans*, i.e., physical damage during pipetting, could have some influence on the measured concentrations, as also mentioned Frangópulos et al. [34]. Nonetheless, this factor would affect controls and mixed cultures in the same way. Moreover, it should be stressed that *N. scintillans* cells in this work were isolated from a dense surface patch in a monospecific red tide, while other studies collected *N. scintillans* from 10 m deep before reaching bloom densities in surface waters [38]. The likely different physiological conditions in both cases (active feeding promotes vacuole production and sinking and starved cells produce ammonia and accumulate in surface waters [36,45], eventually leading to red tides) will probably have some influence on the experimental results.

In conclusion, the dynamics of *N. scintillans* and the toxic dinoflagellate prey in the present study were ruled by negative allelopathic effects, which prevailed over grazing. They occurred in both directions between *N. scintillans* and both *A. minutum* strains, while only from *N. scintillans* towards *D. acuminata*. The lytic effects induced by *A. minutum* correlate with knowledge about the release of BECs in most *Alexandrium* species and with the fact that massive outbreaks of this species, also in the studied region, are nearly monospecific [21]. The absence of this effect in *D. acuminata* and the concomitant increase in DSTs in the presence of *N. scintillans* would deserve further exploration given their putative roles as defense mechanisms. Together, the results in the present study highlight the importance of considering biotic interactions to understand the adaptive traits in harmful algae related with toxins and allelochemicals which confer competitive advantages during the growth season.

## 4. Materials and Methods

*Noctiluca scintillans* cells were collected during a summer red tide in September 2021 from Areamilla beach (Cangas, Ría de Vigo, Spain) using 5 L plastic flasks and transported to the Oceanographic Center of Vigo (IEO-CSIC) in a cooler. In the laboratory, cells were harvested with a Pasteur pipette from the surface orange layer (with only heavily concentrated *N. scintillans* distinguished under the light microscope). These concentrates were rinsed with fresh L1 medium ([45]; without silicates, L1-Si; salinity of 34), onto a 77 µm mesh size sieve to eliminate smaller accompanying organisms, which was corroborated under the light microscope. *Noctiluca scintillans* isolates were maintained in culture chambers at 19 °C with a 12:12 L:D photoperiod and 90 µmol photons m^−2^ s^−1^ irradiance.

*Noctiluca scintillans* cultures were previously concentrated for the experiments to avoid major pipetting errors. Cells were poured onto 10 µm nylon filters (47 mm, Merck, Millipore, Ireland), and resuspended in L1-Si in a 250 mL Erlenmeyer flask (92 cells mL^−1^). Then, *N. scintillans* cells were distributed across several 50 mL Erlenmeyer flasks at a concentration of 20 cells mL^−1^.

One strain of *D. acuminata* (VGO1465) and two strains of *A. minutum* (VGO1435 and VGO1439) obtained from the CCVIEO culture collection (IEO-CSIC, Vigo) were used for the experiments with *N. scintillans* as described below. These species were chosen as representatives of DSP and PSP events in the NW Iberian Peninsula. The strains were grown in L1/20 medium and L1-Si, for *D. acuminata* and *A. minutum*, respectively, and maintained under the same conditions as *N. scintillans*. Cells required for the experimental assays were achieved from exponentially growing cultures.

### 4.1. Experimental Design

The experimental design included triplicate control cultures of each studied strain, *N. scintillans*, *D. acuminata* (VGO1465) and *A. minutum* (VGO1435 and VGO1439), at three concentration levels: 10, 50 and 100 cells mL^−1^ for *D. acuminata*; 100, 500 and 1000 cells mL^−1^ for *A. minutum* strains. Cell densities were within the usual range observed in the field (NW Iberian Peninsula) during the proliferations of these species in the growth season. Maximum cell densities under particular conditions can be higher but were not considered in the experimental design [21,46]. In the case of the *Dinophysis acuminata* complex (including *D. ovum* and *D. sacculus* [46]), bloom densities (10^6^ cells L^−1^) are never observed. Instead, it causes toxic outbreaks at concentrations ranging between 10^2^ and 10^4^ cells L^−1^ [47]. In turn, *Alexandrium minutum* attains maximum concentrations around 10^5^–10^6^ cells L^−1^ [7,21], but during exceptional red tides, such as those in summer 2018, it surpassed 10^7^ cells L^−1^ in confined areas [21].

The concentration of *N. scintillans* was set at 20 cells mL^−1^ in controls and all mixed cultures. In addition, *N. scintillans* cultures were exposed to a cell-free filtrate of *A. minutum* VGO1435 to discern any effects which could be associated with released compounds in the medium.

For cell-free filtrate experiment, cells from VGO1439 strain were retained on a glass microfiber filter MFV3 (47 mm). Then, cell-free L1 medium was distributed in the different flasks using the same volumes and procedures as in the mixed culture experiment.

All the experiments were conducted in triplicate. Incubations were carried out in Biolite 50 mL culture flasks (Thermo Fisher Sci., New York, NY, USA) over 4 days at 19 °C. Daily cell counts were performed using 1 mL aliquots from each culture flasks and fixed with acid Lugol’s iodine solution (approx. 0.5% final concentration). Samples were counted using an inverted microscope (Nikon Eclipse TE2000-S, Minato, Japan) at a magnification of 100×, with a Sedgewick Rafter Chamber [48].

### 4.2. Toxins

Preliminary analyses of PSP and DSP toxins in the studied strains of *A. minutum* and *D. acuminata* were carried out in exponentially growing cultures before launching the experiments to confirm their toxin profiles and quantify toxin cell content. Samples for toxin analyses were collected from each one of the experiments on day 4. LODs of the toxins analyzed were calculated using the standard deviation of the response (Sy) and the slope (S) of the calibration curve according to the formula LOD = 3.3 (Sy/S).

#### 4.2.1. *Dinophysis acuminata*: OA Extraction and Detection

At the end of the experiment (day 4), controls were filtered by glass microfiber filters MFV3 (25 mm). Mixed cultures were sieved onto a 77 µm mesh size and subsequently filtered by glass microfiber filters MFV3 (25 mm) to separate *N. scintillans* and *D. acuminata.* *D. acuminata* cultures were filtered by glass microfiber filters MFV3 (25 mm). DSTs contained in *D. acuminata* and *N. scintillans* cells were extracted twice with 750 µL of MeOH 100%, filters were sonicated for 15 s with an Ultrasonic Homogenizer 4710 series (Cole-Parmer, Chicago, IL, USA) and centrifuged at 17,968× *g* and 10 °C for 10 min (Sigma 3-16 KL, Sartorius, Osterode am Harz, Germany). Both extracts were combined (1500 µL final volume) and kept in the fridge at −20 °C until toxin analyses. Extracts were filtered by hydrophilic PTFE 0.22 µm syringe filters before chromatographic analysis. DSTs contained in the culture filtrates were extracted by solid-phase extraction (SPE) with Sep-Pak C18 light cartridges (Waters, Wexford, Ireland) using a Manifold coupled to a vacuum pump (Merck Millipore, Darmstadt, Germany). Cartridges were conditioned with 3 mL of MeOH 100% and 3 mL of MQ water. Then, a 40 mL sample was loaded. The cartridge was cleaned with 4 mL of MeOH 20% to remove salts and elution of toxins was taken with 4 mL of MeOH 100%.

Lipophilic toxins were identified by liquid chromatography coupled to high-resolution mass spectrometry (LC-HRMS), with a Dionex Ultimate 3000 LC system (Thermo Fisher Scientific, San-José, CA, USA) coupled to an Exactive mass spectrometer (Thermo Fisher Scientific, Bremen, Germany) equipped with an Orbitrap mass analyzer and a heated electrospray source (H-ESI II). Nitrogen (purity > 99.999%) was used as the sheath gas and auxiliary gas. The instrument was calibrated in positive and negative ion modes. The mass range was *m*/*z* 100–1200 in negative full-scan mode and *m*/*z* 100–1000 in positive mode. 

OA was separated and quantified according to the standardized operating procedure validated by the European Union Reference Laboratory for Marine Biotoxins (EURLMB, 2011). Toxins were separated in a Gemini NX-C18 column (100 × 2 mm, 3 µm) maintained at 40 °C; the injection volume was 20 µL and the flow rate was 0.4 mL min^−1^. Mobile phase A was MQ water, and mobile phase B was a mix of acetonitrile/MQ water (90/10 *v*/*v*), both containing 0.05 M *v*/*v* % ammonia. The process was a linear gradient, with 25% of B (from 0 to 1.5 min), 95% of B (from 7.5 to 9.5 min) and 25% of mobile phase B was restored from 12.5 min to 17 min. A mixture of OA, DTX2, DTX1 and PTX2 standards (concentration ranged between 5 and 40 ng mL^−1^) was used and acquired from CIFGA laboratories, Lugo, Spain.

#### 4.2.2. *Alexandrium Minutum*: PSP Toxins’ Extraction and Detection

The same initial procedure as in the case of *D. acuminata* was followed. Controls were filtered by glass microfiber filters MFV3 (25 mm) and mixed cultures were sifted by gravity using a 77 µm nylon mesh to separate *N. scintillans* from *A. minutum*. Afterward, *A. minutum* cultures and mixed cultures were filtered by glass microfiber filters MFV3 (25 mm). PSTs contained in *A. minutum* cells and in *N. scintillans* were extracted twice with 750 µL of acetic acid 0.05 M, filters were sonicated for 15 s with a sonication probe and centrifugated at 17,968× *g* and 10 °C for 10 min (final volume 1500 µL^−1^). The extracts were kept at −20 °C until LC analyses and then filtered through hydrophilic PTFE 0.22 µm syringe filters. Dissolved PSTs contained in the culture filtrates were extracted by solid-phase extraction (SPE) with Sep-Pak Vac 3cc tC18 Cartridges (Waters, Ireland, Wilmslow, UK) using a Manifold under vacuum pressure according to Boundy et al. [49]. Cartridges were conditioned with 3 mL of acetonitrile/water/acetic acid (20:80:1 *v*/*v*/*v*) and 3 mL of MQ water/NH_4_OH (1000:1 *v*/*v*). Then, 400 µL of sample extract was loaded. The cartridge was cleaned with 700 µL of MQ water and toxins were eluted with 2 mL of MeCN/MQ/acetic acid (20:80:1).

PSP toxins were analyzed by liquid chromatography with fluorescence detection and post-column oxidation (LC-FLD-PCOX) following Rodríguez et al. [50]. A mixture of GTX4, GTX1, GTX3 and GTX2 standards (concentration ranged between 0.0149 and 7.525 ng µL^−1^) was used and acquired from CIFGA laboratories, Spain.

### 4.3. Growth

Specific growth rates (μ, day^−1^) were estimated following Guillard [51]:

(1) $$\mu =(lnN_{\left(i+1\right)}-lnN_{i})/(t_{\left(i+1\right)}-t_{i})$$

where *N_i_* and *N*_(*i*+1)_ are cell densities (cells mL^−1^) at time *t_i_* and *t*_(*i*+1)_ (days).

### 4.4. Statistical Analysis

Statistical analyses to determine the effects of different cell concentrations and species were performed by comparing analysis of variance (ANOVA). Homogeneity of variances was tested using *Levene* test. Whenever the analysis of variance assumption was violated, data were rank-transformed to create a uniform distribution. Homogeneous groups were established a posteriori using the Tukey test for pair-wise comparisons among treatments. These analyses were performed using the STATISTICA 7.0 software (Tulsa, OK, USA).

## Figures and Tables

**Table 1:** *Dinophysis acuminata* (VGO1465) final total cells and OA concentrations in control and mixed cultures at the end of experiment (day 4). Initial abundance: 10, 50 and 100 cells mL^−1^. Data represent mean (*n* = 3) and error bars correspond to the SD. Limit of detection (LOD) for OA was 0.03 ng on column.

	*D. acuminata* (VGO 1465) (Controls)			*D. acuminata* (VGO 1465) (Mixed Cultures)		
Initial abundance (cells mL^−1^)	10	50	100	10	50	100
Final total cells (day 4)	13 ± 2.08	82 ± 14	164 ± 7	3 ± 2	13 ± 5	37 ± 8
Intracellular OA (pg cell^−1^)	<LOD	57.7 ± 9.94	46.6 ± 12.69	<LOD	<LOD	61.28 ± 9.46
OA in the culture-filtrate (ng mL^−1^)	<LOD	1.17 ± 0.00	1.93 ± 0.20	<LOD	1.73 ± 0.04	2.37 ± 0.31

**Table 2:** Total cells of *A. minutum* (VGO1435) in controls and PST concentrations at the three abundances assayed (100, 500 and 1000 cells mL^−1^) at the end of the experiment (day 4). Data represent mean (*n* = 3) and error bars correspond to SD. LODs (ng on column): 1.8 (GTX4); 3.8 (GTX1); 0.2 (GTX3); and 0.1 (GTX2).

	*A. minutum* (VGO1435) (Controls)		
Initial abundance (cells mL^−1^)	100	500	1000
Final total cells (day 4)	3.33 (±0.14) × 10^4^	1.43 (±0.2) × 10^5^	3.62 (±0.86) × 10^5^
pg STX equivalents cell^−1^ (day 4)	2.27	2.64	1.89
GTX4 (pg cell^−1^)/(molar %)	2.75 ± 0.14/85	3.05 ± 0.34/81	2.35 ± 0.38/87
GTX1 (pg cell^−1^)/(molar %)	0.15 ± 0.05/7	0.20 ± 0.03/8	0.07 ± 0.04/ 4
GTX3 (pg cell^−1^)/(molar %)	0.29 ± 0.01/8	0.46 ± 0.05/11	0.25 ± 0.05/8
GTX2 (pg cell^−1^)/(molar %)	0.04 ± 0.02/1	0.07 ± 0.01/1	0.05 ± 0.00/1

**Table 3:** Total cells and PST concentrations in control and mixed cultures of *A. minutum* (VGO1439) strain at the end of experiment (day 4). Concentrations assayed: 100, 500 and 1000 cells mL^−1^. Data represent mean (*n* = 3) and error bars correspond to the SD. LODs (ng on column) 1.8 (GTX4); 3.8 (GTX1); 0.2 (GTX3); and 0.1 (GTX2). PSTs at 100 cells mL^−1^ were <LOD.

	*A. Minutum* (VGO1439) (Controls)		*A. Minutum* (VGO1439) (Mixed Cultures)	
Initial abundance (cells mL^−1^)	500	1000	500	1000
Final total cells (day 4)	10.27 (±1.15) × 10^4^	26.02 (±4.2) × 10^4^	19.23 (±1.16) × 10^3^	31.47 (±1.6) × 10^3^
pg STX equivalents cell^−1^	1.26	1.21	0.03	0.03
GTX4 (pg cell^−1^)/(molar %)	1.30 ± 0.46/71	1.24 ± 0.27/72	0.04 ± 0.00/ 100	0.02 ± 0.00/41
GTX1 (pg cell^−1^)/(molar %)	0.15 ± 0.03/12	0.15 ± 0.04/12	<LOD	0.02 ± 0.00/59
GTX3 (pg cell^−1^)/(molar %)	0.27 ± 0.06/13	0.26 ± 0.07/13	<LOD	<LOD
GTX2 (pg cell^−1^)/(molar %)	0.10 ± 0.01/ 3	0.08 ± 0.02/3	<LOD	<LOD

## Data Availability

Not applicable.
